# Beta-globin gene haplotypes and selected Malaria-associated variants among black Southern African populations

**DOI:** 10.1017/gheg.2017.14

**Published:** 2017-11-27

**Authors:** G. D. Pule, E. R. Chimusa, K. Mnika, K. Mhandire, E. Kampira, C. Dandara, A. Wonkam

**Affiliations:** 1 Division of Human Genetics, Department of Pathology, Faculty of Health Sciences, University of Cape Town, Cape Town, South Africa; 2 Departments of Chemical Pathology, University of Zimbabwe, Harare, Zimbabwe; 3 Malawi College of Health Sciences, University of Malawi, Blantyre, Malawi

**Keywords:** *β*-globin haplotypes, malaria resistance, sickle cell disease, Southern Africa

## Abstract

Partial carrier-resistance to *Plasmodium falciparum* malaria conferred by the sickle cell (*HbS*) mutation has resulted in the local amplification and positive selection of sickle cell disease (SCD) in malaria-endemic regions and particularly in sub-Saharan Africa (SSA). The present study investigated the *β*-globin gene haplotypes, and selected malaria-associated variants among three cohorts of Bantu-speaking individuals from Malawi, Zimbabwe and South Africa compared with reports with data from others SSA populations. The data suggest a south-ward frequency decrease of malaria-associated variants in SSA linked to the evolutionary dynamics of various African populations’ genomes through selective pressure of malaria. These selected genomics differences, positive selection of SCD in malaria-endemic regions among ‘Bantus’ from various part of Africa emphasise the evidence of the dissociation between genetics, anthropology and culture. The present study also showed a relatively prevalent Benin haplotype, which is mostly found in West Africa, among Southern African Blacks and very low Bantu haplotype, which could suggest a major migration route, of Southern Africa Bantu, along the African west coast, post-occurrence of the Sickle cell mutation, which date remain to be fully elucidated.

## Introduction

Sickle cell disease (SCD) is a monogenic, hematological and multi-organ disorder affecting the structure of erythrocytes by altering the normal biconcave shape to a crescent [1]. The sickling results from the polymerization and precipitation of the *β*-globin chains (*HbS*) during deoxygenation and dehydration of erythrocytes [2]. The vascular pathology of the disease includes platelet and leukocyte adhesion abnormality and hypercoagulation leading to microvascular occlusion, hemolysis and hypoxia and ultimately, multi-organ damage.

There is a strong correlation between the frequency of the HbS gene and the historical distribution and incidences of malaria [3] because of the partial carrier-resistance to *Plasmodium falciparum* malaria. The geographical co-occurrence of SCD and malaria and the partial carrier-resistance is believed to have resulted in the local amplification and positive selection of SCD in malaria-endemic regions [4, 5]. A GWAS for severe malaria in Ghana and the Gambia reported four loci with genome-wide significant single-nucleotide polymorphisms (SNPs) associated with the disease. Two of them were tag SNPs of previously known causal variants (*rs8176703* in *ABO*, causal variant *rs8176719*; *rs372091* in *HBB*, causal variant *rs334*), whereas the other two were novel loci with unknown causal SNPs. The ABO locus has the previous indication of a protective effect conferred by the blood group O against severe malaria [6–8]. The variant *rs2334880*, was one of the novel resistance loci identified and was mapped to 6.4 kb upstream of the MARVEL domain-containing protein 3 gene (*MARVELD3*; MIM ID*614094), which forms part of multiple tight-junction of epithelial and vascular endothelial cells [9–11] and is strongly associated with severe malaria [12]. It is, however, noteworthy that no function mutation at *MARVELD3* is known and, in the current literature, evidence of association is conflicting [5, 6, 10]. The endemicity of malaria in sub-Saharan Africa (SSA) and the associated *HbS* mutations, has resulted in the highest SCD burden with nearly 80% of the approximately 300 000 new affected births that occur in SSA annually [13].

The *HbS* mutation is believed to have evolved independently in five regions of the world, classically associated with five region-defined haplotypes, four of which are African, based on conserved patterns of polymorphisms across the *β*-globin gene cluster, namely Benin, Central African (CAR) or Bantu; Cameroon; Senegal and Indian-Arab [4, 14, 15]. A recent review of the global distribution and frequencies of these haplotypes has provided a glimpse into population dynamics and migration within and out of Africa that has prompted the hypothesis of a single origin of *HbS* mutation [16]. In this context, the study of malaria associated variants among Southern African populations, specifically among South African Blacks that have been living outside the malaria-endemic equatorial belt for 3–5000 years [17, 18], could provide new insight into the within-Africa migration patterns, and some perspectives into the dissociation between genetics and anthropology, with regard to differential allele frequencies related to various conditions such as malaria, susceptibility and resistance among Bantu-speaking groups from various parts of Africa.

In this present study, we investigated the *β*-globin gene haplotypes and selected malaria-associated variants among three cohorts of healthy Southern African populations from Malawi, Zimbabwe and South Africa and compared the frequencies of these variants to that of other SSA populations, and data extracted from the 1000 Genome Project.

## Methods

### Ethics approval

The study was performed with the approval of the University of Cape Town, Faculty of Health Sciences Human Research Ethics Committee (HREC REF: 132/2010 and HREC REF: 1094/2009).

### Populations

A total of 158 DNA samples (50 Zimbabweans; 58 Malawians and 50 South Africans) all of Bantu origin were randomly selected for the Division of Human Genetics bio-repositories, Faculty of Health Sciences, University of Cape Town. These participants were randomly sampled from a cohort of the unrelated and apparently healthy individual, initially recruited for a population genetics study.

## Genotyping

### HbS mutation and *β*-globin haplotypes

Using the participants from the three southern African populations, PCR and Dde I restriction analysis were used to confirm the absence of the HbS mutation [19] and published primers and methods [20] genotyping five restriction fragment length polymorphic (RFLP) regions in the *β*-globin gene cluster were used to analyse the XmnI (5'Gγ), HindIII (Gγ), HindIII (Aγ), HincII (3˙’Ψ*β*) and HinfI (5’*β*) loci for the HbS haplotype background (online Supplementary Table S1) [16]. Restriction endonuclease cutting patterns that represent each of the five most common atypical *β*-globin gene haplotypes are represented in online Supplementary Table S2.

### Selection of Malaria associated SNPs

To compare the Minor Allele Frequencies (MAF) of malaria-associated SNPs between African living outside (mainly our three cohorts) and the malaria-endemic equatorial populations. We selected among recently identified malaria SNPs in [21], SNPs under linkage equilibrium, mostly with a great number of LD proxy variants in both Western (YRI, Yoruba) and eastern (LWK, Luhya in Webuye, Kenya) African Bantu. In doing so, three SNPs include *rs8176703*, *rs372091* and *rs2334880* that meet the above criteria (online Supplementary Figs S1–S3). These three SNPs are in fairly low LD (*r*^2^ < 0.2) with the primary functional mutations [1], but being the most-associated markers in the GWAS conducted in [21]. To genotype these targeted SNPs, SNaPshot multiplex genotyping (based on the incorporation of a single ddNTP to an extension primer designed to anneal 1 bp upstream of the target SNP), and followed by capillary electrophoresis were used, according to a previously reported method [22]. Up to 10% of the genotypes’ results were confirmed, by direct Sanger sequencing.

### Data analysis and bioinformatics analysis using data extracted from the 1000G

Genotyping at the characterised loci conformed to Hardy–Weinberg Equilibrium (HWE) (*p* values > 0.05). Leveraging the moderated sample size and the accurate publicly phased data from 1000 Genomes Project, we compared the MAF of the selected SNPs to those of other African and non-African populations, and analysed the diversity of the beta-globin haplotype in five other African populations. We have used a custom python script to extract the data of five African populations from 1000 Genome project phase3 on chromosome 11 in a 100 kb region around *HBB*. The data included 108 samples from Yoruba (YRI) in Nigeria, 99 from Esan (ESN) in Nigeria, 113 from Gambia (GWD) in Western Divisions in the Gambia, 99 Luhya (LWK) in Webuye, Kenya and 85 from Mende (MSL) in Sierra Leone. Plink software [23] was used to compute the haplotype blocks in each of those populations. Each inferred haplotype blocks was utilised in plink to estimate the haplotype frequency within the specific population. Similarly, the LD blocks were computed using Plink based on LD r2, and the LD pattern was visualised using Haploview [24]. From a custom R script, we have made use of 20 haplotypes from each population to plot the haplotype bifurcation at the variant *rs334*.

## Results

### Sickle cell genotype frequencies

The description of the HbS allele frequency, *β*-globin haplotype background and selected malaria-related SNPs for the study cohorts are given in Table 1. All participants from South Africa (100%, *n* = 50); and the majority from Zimbabwe (88%, *n* = 50) and Malawi (93.5%, *n* = 58) were determined to be homozygous unaffected (HbAA), with the rest being heterozygous for the sickle mutation (HbAS).

**Table 1.** Frequencies of the HbAA; *β*-globin haplotypes and malaria-related SNPs

		South Africa *N* (%)	Zimbabwe *N* (%)	Malawi *N* (%)
*β*-globin mutation	HbAA	50 (100)	44 (88.0)	58 (93.5)
	HbAS	0 (0.0)	6 (12.0)	4 (6.5)
*β*-globin haplotypes^a^	Atypical	53 (68.0)	42 (65.7)	36 (51.4)
	Benin	13 (16.6)	8 (12.5)	19 (27.1)
	Bantu	4 (5.1)	2 (3.1)	4 (5.7)
	Cameroon	5 (6.4)	10 (15.6)	5 (7.1)
	Senegal	3 (3.9)	2 (3.1)	6 (8.7)
*β*-globin haplotype recombinants^b^	Atypical/Atypical	16 (41.0)	12 (38.0)	8 (23.5)
	Benin/Atypical	13 (33.3)	8 (25.0)	14 (41.2)
	Bantu/Atypical	4 (10.3)	2 (6.3)	2 (5.9)
	Senegal/Atypical	2 (5.1)	1 (3.1)	4 (11.8)
*rs8176703*	GG	35 (0.97)	48 (0.96)	48 (0.98)
	AG	1 (0.03)	2 (0.04)	1 (0.02)
	AA	0 (0.0)	0 (0.0)	0 (0.0)
*rs372091*	GG	34 (0.94)	42 (0.93)	48 (0.98)
	AG	1 (0.03)	3 (0.07)	1 (0.02)
	AA	1 (0.03)	0 (0.0)	0 (0.0)
*rs2334880*	CC	7 (0.23)	10 (0.21)	14 (0.29)
	CT	19 (0.63)	23 (0.48)	24 (0.50)
	TT	4 (0.13)	15 (0.31)	10 (0.21)

a *β*-globin haplotype frequencies are given as the number of chromosomes presenting with a specific haplotype.

b *β*-globin haplotype recombinants: the pair of haplotypes inherited in two separate chromosomes in an individual.

### Haplotypes in the *β*-globin gene cluster

**Fig. 1.**
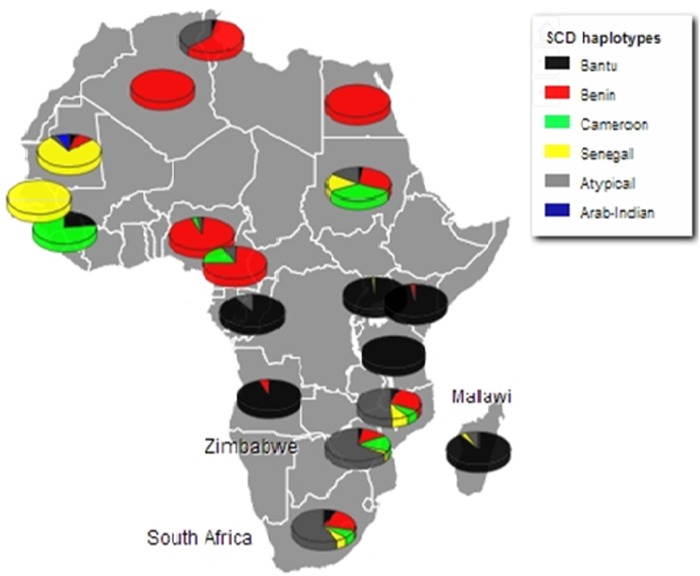
Population frequencies of all haplotypes for the study cohorts (South Africa, Zimbabwe and Malawi), versus frequencies conditional on being homozygous for HbS in the other population's groups across the continent. (with adaptation of previously reported from [16]).

SCD exists in Africa on disparate haplotype backgrounds [25] and is described by a specific pattern of five SNPs across the *β*-globin gene cluster [16]. This pattern confers four haplotypes associated with the HbS mutation in Africa; Benin, Bantu/Central African Republic (CAR), Senegal and Cameroon, with the fifth haplotype arising in the Indian/Arabian peninsula (Arab/Hindu) [15, 26]. Any recombination of the defining SNPs results in recombinant haplotypes referred to as ‘atypical’. The SCD haplotypes were described using a previously published method and the global distribution of the haplotypes reviewed [16]. The haplotypes were described based on the analysis of chromosomes from the South Africa, Zimbabwe and Malawi cohorts (78, 64 and 70 chromosomes respectively), the most prevalent of the *β*-globin gene haplotypes was the atypical form; 67.9, 65.6 and 51.4%, respectively. Specifically, atypical I was common across all three populations at similar frequencies, (32.1% South Africa; 38.1% Zimbabwe and 38.9% Malawi) (online Supplementary Table S3). The two second most prevalent haplotypes were the Benin and Cameroon forms. In combination, the atypical/atypical haplotype was most frequent in the South Africa and Zimbabwe cohorts (41.0 and 37.5%, respectively) whereas the Benin/atypical was the most frequent combination in Malawi (41.2%). Figure 1 shows the distribution of the SCD *β*-globin gene haplotypes amongst the study cohorts compared with the haplotypes reported in SCD patients in other African countries [16].

### Targeted Malaria-related variants

**Fig. 2.**
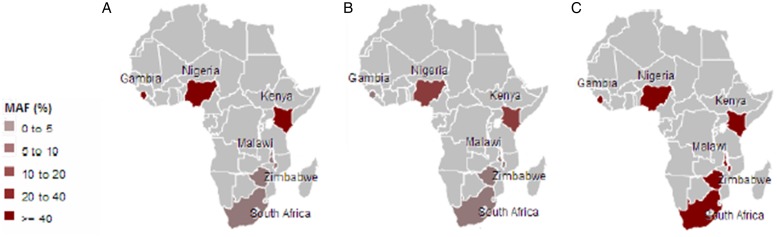
Minor frequencies of malaria-restriction SNPs amongst southern African populations and three populations from the 1000Genomes Project within the malaria-endemic central Africa. A: *rs8176703*; B: *rs372091*; C: *rs2334880*.

**Fig. 3.**
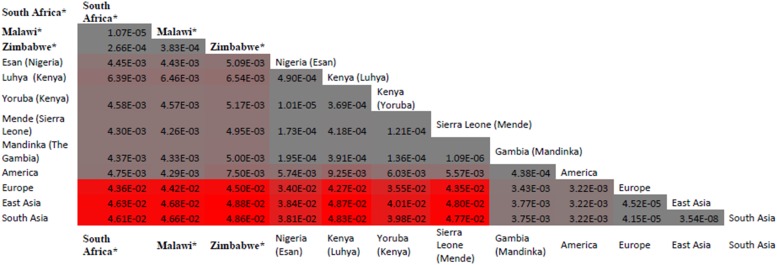
Distribution of frequency differentiation of targeted SNPs *rs8176703*; *rs372091* and *rs2334880* across various African populations. When comparing the measure of frequency differentiation among the genotyped SNPs and the corresponding frequencies of these SNPs in the 1000Genomes data, the frequency of the genotyped SNPs were highest among the Southern African populations and the African populations (Esan, Luhya, Yoruba, Mende and Mandinka) (Fig. 3). The frequencies were lowest between American, Asian and European populations. * Populations studied in from the current paper (South Africa, Zimbabwe and Malawi); other data were extracted from the 1000G project. The values provided are F-statistics calculated between each MAF for the three SNPs (*rs8176703*; *rs372091* and *rs2334880*) and colored coded grey (genetically proximal) to red (genetically distal). Populations with less genetic distance have lower F-st and shown in grey whereas populations with greater genetic distance have higher F-st and are shown in red.

Malaria has slight low incidence in Southern compare with Western-central (equatorial region) Africa and given that sickle cell anemia patients are known for potential resistance to the parasite that causes malaria [1]; it is therefore worth to investigate the population allele frequency at these resistance loci between populations in Southern and Western-central Africa. After discarding associated variants under LD and prioritizing associated variants with high proxy LD variants, three SNPs were selected (online Supplementary Figs S1–S3); *rs8176703* (9q34.2; ABO), *rs2334880* (16q22.2; *MARVELD3*) and *rs372091* (11p15.5; *HBB*) from the GWAS results in [21], to probe the allele frequencies and relative geographic distribution around and below the equatorial malaria belt. Despite the conflict in literature regarding the role of *MARVELD3*, this approach was driven by the hypothesis that, such resistance loci even at the level of single nucleotide polymorphisms, which confer clinically significant resistance to severe malaria would undergo strong positive selection in malaria-endemic regions and to a gradual lesser extent, regions around the equatorial belt. Therefore, the allele frequencies of three variants at resistance loci [21] were investigated among three sub-Saharan African populations (Malawi, Zimbabwe and South Africa) at varying proximity to the equatorial malaria endemicity belt. SCD unaffected populations were selected in order to eliminate the possible effect of co-inheritance of malaria resistance loci and the *HbS* allele, as a result of the HbS allele-conferred partial resistance to *P. falciparum.* The genotype frequencies for the *rs8176703* (GG), *rs372091* (GG) and *rs2334880* (CT) among South African, Malawian and Zimbabwean populations were largely similar. However, when comparing MAFs at these loci with other populations from the Human 1000 Genome Project, 1000 Genomes Phase III, there was an apparent gradient of the MAF for *rs8176703* and *rs372091*, highest in countries within the equatorial malaria belt (Gambia, Nigeria and Kenya) and lowest in the sub-equatorial populations investigated in this study (Table 2, Fig. 2). However, this pattern was not observed in the MAF at *rs2334880*. When comparing the measure of frequency differentiation among the genotyped SNPs and the corresponding frequencies of these SNPs in the 1000 Genomes data, the frequency of the genotyped SNPs were highest among the Southern African populations and the African populations extracted from the 1000 Genomes Project (Esan, Luhya, Yoruba, Mende and Mandinka) (Fig. 3). As expected, the frequencies were lowest among American, East-Asian and European populations, consistent with the fact that these geographic regions do not have a problem with malaria, and that the incidence of sickle cell anemia is decreasing [27]. Table 3 and online Supplementary Fig. S4 show the frequency of the *HbS* allele across African populations [13, 27–36]. When investigating the LD between these variants in the African 1000 Genomes phase3 data, these variants were found to be in linkage equilibrium with their respective functional mutations, suggesting deep sequencing to potentially prioritise novel mutation variants.

**Table 2.** Minor allele frequencies of study cohorts and several populations from the 1000Genomes Project

Region	Variants		
	rs8176703	rs372091	rs2334880
South Africa^a^	0.013	0.023	0.472
Malawi^a^	0.01	0.01	0.457
Zimbabwe^a^	0.02	0.033	0.552
African	0.398	0.068	0.398
African Caribbean (Barbados)	0.359	0.047	0.359
Southwest US (African American)	0.311	0.041	0.311
Nigeria (Esan)	0.399	0.141	0.399
Kenya (Luhya)	0.470	0.071	0.470
Kenya (Yoruba)	0.407	0.125	0.407
Mende (Sierra Leone)	0.400	0.024	0.400
Gambia (Mandinka)	0.403	0.013	0.403
America	0.102	0.001	0.102
Europe	0.160	1.000	0.160
East Asia	0.009	1.000	0.009
South Asia	0.019	1.000	0.019

a Study populations from the current study. Other data was sourced from the 1000G project.

**Table 3.** HbS allele frequencies by country in Africa

Country	Study years	Population	Age group	HbS allele frequency	Reference
Angola	1950–2010	18 994	All ages	0.137	[13]
Benin	1950–2010	9219	All ages	0.159	[13]
Botswana	1950–2010	1977	All ages	0.003	[13]
Burkina Faso	1950–2010	16 250	All ages	0.056	[13, 30, 31]
	1997–1999	9201	New-borns	0.0025	
			Median 9 years	0.0013	
Burundi	1950–2010	8519	All ages	0.040	[13]
Cameroon	1950–2010	19 957	All ages	0.120	[13]
Cape Verde	1950–2010	513	All ages	0.026	[13]
Central African Republic	1950–2010	4506	All ages	0.077	[13]
Chad	1950–2010	11 509	All ages	0.051	[13]
Comoros	1950–2010	691	All ages	0.018	[13]
Congo	1950–2010	3760	All ages	0.145	[13]
Democratic Republic of the Congo	1950–2010	67 829	All ages	0.165	[13]
Djibouti	1950–2010	879	All ages	0.001	[13]
Equatorial Guinea	1950–2010	693	All ages	0.192	[13]
Eritrea	1950–2010	5204	All ages	0.003	[13]
Ethiopia	1950–2010	84 996	All ages	0.003	[13]
Gabon	1950–2010	1501	All ages	0.280	[13]
Gambia	1950–2010	1751	All ages	0.075	[13, 29]
	2003	536	New-borns	0.012	
			10–72 months	0.003	
Ghana	1950–2010	24 339	All ages	0.087	[13]
	2002	1266	0–4 years	0.0039	
		842	5–10 years	0.0012	
Guinea	1950–2010	10 324	All ages	0.168	[13]
Guinea-Bissau	1950–2010	1647	All ages	0.041	[13]
Kenya	1950–2010	40 835	All ages	0.038	[13, 15, 28]
	1998–1999	2774	New-borns	0.016	
		782	0–3 years	0.006	
	1998–2008	282	0–11 months	0.01	
		415	12–23 months	0.0035	
		3677	3–5 years	0.0024	
			6–13 years	0.0009	
Lesotho	1950–2010	2064	All ages	0.001	[13]
Liberia	1950–2010	4102	All ages	0.046	[13]
Madagascar	1950–2010	20 146	All ages	0.061	[13]
Malawi	1950–2010	15 690	All ages	0.033	[13]
Mali	1950–2010	13 362	All ages	0.057	[13]
Mauritania	1950–2010	3359	All ages	0.050	[13]
Mozambique	1950–2010	23 418	All ages	0.027	[13]
Namibia	1950–2010	2212	All ages	0.010	[13]
Niger	1950–2010	15 885	All ages	0.080	[13]
Nigeria	1950–2010	158 255	All ages	0.171	[13, 26]
	1970–1972	534	New-borns	0.021	
		259	1–4 years	0.004	
		637	5–14 years	0.002	
Rwanda	1950–2010	10 277	All ages	0.023	[13]
Sao Tome and Principe	1950–2010	165	All ages	0.094	[13]
Senegal	1950–2010	12 866	All ages	0.067	[13, 24, 25]
Senegal (rural kegoudou)	2002–2003	432	New-borns	0.005	
			2–10 years	None	
			Newborn	0.01	
Sierra Leone	1950–2010	5837	All ages	0.164	[13]
South Africa	1950–2010	50 523	All ages	0.003	[13]
Sudan	1950–2010	43 182	All ages	0.043	[13]
Swaziland	1950–2010	1195	All ages	0.006	[13]
Tanzania, United Republic of	1950–2010	45 028	All ages	0.074	[13]
Uganda	1950–2010	33 798	All ages	0.082	[13]
Zambia	1950–2010	13 254	All ages	0.112	[13, 23]
	1967–1971	2845	0–11 months	0.013	
		2200	1–3 years	0.009	
		2306	3–12 years	0.005	
Zimbabwe	1950–2010	12 645	All ages	0.021	[13]

### Pattern of linkage disequilibrium and haplotype blocks at *rs334* in African populations

**Fig. 4.**
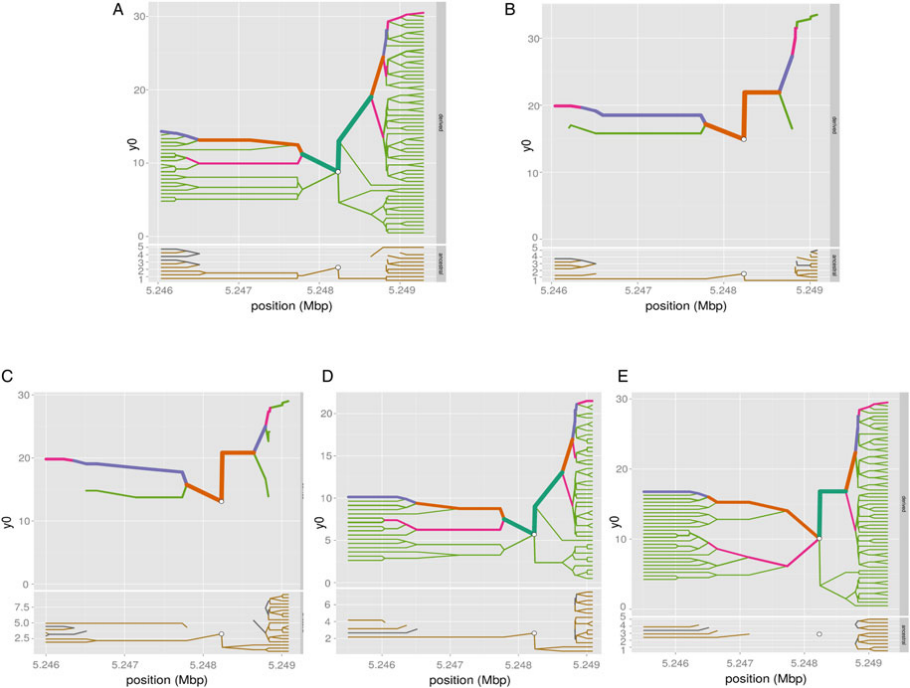
Haplotype bifurcation diagrams. The root of each diagram is a core haplotype at the variant *rs334*, identified by a white circle. The diagram is bi-directional, portraying both proximal and distal LD for derived (each top) and ancestral allele (each bottom). The breakdown of LD on the core haplotype background is portrayed at progressively longer distances, depending on whether the allele is present or not. The thickness of the lines corresponds to the number of samples with the indicated long-distance haplotype. (A) ESN (B) YRI (C) GWD (D) MSL and (E) LWK.

**Fig. 5.**
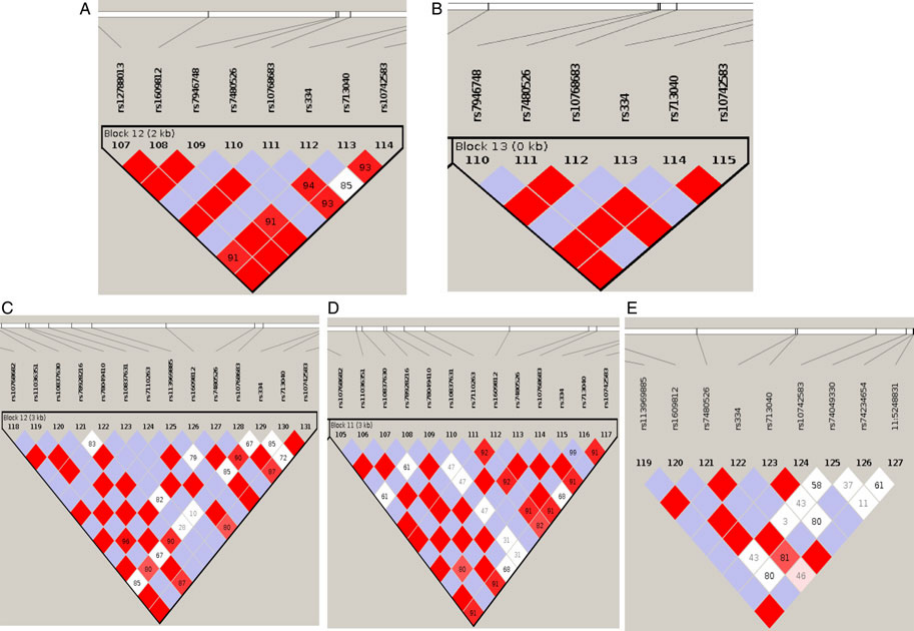
Pattern of linkage disequilibrium. A linkage disequilibrium (LD) block of polymorphisms in a tight region around *rs334*. (A) ESN (B) YRI (C) GWD (D) MSL and (E) LWK.

We have computed the haplotype blocks, block of linkage disequilibrium and the haplotype frequency in a 100 kb region around *HBB*, targeting the variant *rs334* in that region, which is well known of alleles A/T, encoding the *Hb A* form of (adult) hemoglobin and the sickling form of hemoglobin, *Hb S*, respectively. The results in Figs 4 and 5 show differing pattern of LD between Western and Eastern African Bantu.

## Discussion

The present data confirm the evolutionary dynamics of various African Bantu genomes through selective pressure of malaria, and prompt the persecution of the dissociation between genetics and anthropology and culture, and lastly illustrated the importance of understanding the migration path of southern African populations, as a result of the past 1200 years southern African Bantu migration and various contact with sea-borne immigrants from Europe, Asia and Indonesia [37, 38].

### South-ward frequency decrease of malaria-associated SNPs in SSA

As a result of the known partial resistance conferred by the *HbS* allele to malaria *Plasmodium falciparum* infection, the HbS allele is highly prevalent in malaria-endemic regions particularly around the tropical equatorial belt in SSA [3, 4]. The study confirms the accepted notion of low *HbS* allele frequency in populations outside malaria-endemic regions (online Supplementary Fig. S4; Table 3). In addition to the *HbS* mutation, whose association with malaria is extensively studied, we additionally selected three malaria associated SNPs from GWAS conducted by Timmann *et al.* [21] in linkage-equilibrium and with differentiate level of proxy LD comparing western and eastern African (online Supplementary Figs S1–S3) to compare the population allele frequency between our Southern cohorts and Western-central populations. MAFs of these three variants were determined and compared with those in Gambia, Nigeria and Kenya (1000 Genomes data) and showed a decreasing gradient of MAF for two of the loci (*rs8176703* and *rs372091*), which were highest within the equatorial malaria belt and lowest in all three study cohorts. However, there was no such gradient for the *rs2334880* variant, with similar MAF across all six populations, therefore not specifically linked with malaria endemicity, which is concomitant with results from an independent study where this variant failed to replicate its association [2, 3]. This trend suggests that although all three variants were highlighted with low LD to known functional mutations [1] and have been associated with resistance to severe malaria [21], only two (*rs8176703* and *rs372091*) are largely restricted to the equatorial malaria belt and possibly confer greater resistance to severe malaria as compared with the less equator-bound variant (*rs2334880*). The observed gradient for *rs8176703* and *rs372091* could indeed reflect that these variants are also associated with causal variants outside of West Africa, however, the absence of a gradient for *rs2334880* can just indicate that this variant is a poor proxy for the putative causal variant there. Furthermore, it is noteworthy that the *ATP2B4* [1] and *FREM3* [2] loci were not genotyped in this study due to a limitation of resources and challenges with assay optimization. Furthermore, several other loci have been associated with malaria-resistance and were not included in the present study. Future work should consider determining the frequency and effect of not only LD SNPs but include functional mutations at several loci to improve our understanding of the malaria-resistance genotype profile of Southern African populations, largely unexposed to malaria.

### Haplotype blocks at *rs334* in African populations

The differing pattern of LD between Western and Eastern African Bantu can be explained by the fact that Eastern African Bantu undergone admixture due to the various contacts with sea bone migrants in their past history [37]. However, the result in online Supplementary Table S4 suggests a similar pattern of beta globin haplotype diversity at the variant rs334 across all the five African populations (Western and Eastern Bantu). This illustrates the importance of investigating the origin and age of the *HbS* mutation following the past southern Bantu migration, admixture and population sub-structure.

### Implications for genetics, anthropology and culture: Bantu is not equal to Bantu

Taken in sum; the frequency of *HbS* mutation and the decreasing MAF gradient of the targeted SNPs from the tropical malaria-endemic regions towards the South suggest that the specific combination and pattern of multiple malaria resistant variants could allow the broad determination of the regional origins of an individual as Western, Central or Southern African. Although the vast majority of differentiated loci among Bantu populations are no more differentiated than would be expected from population drift, the modest data presented here support the proposed notion of dissociation between genetic background and ethno-linguistic attributes and classifications. Indeed, there are several indicators of a linguistic and cultural similarity among Bantus; for instance (i) ‘muntu’ for ‘human’ is the same in Xhosa (South African Bantu language) and Ewondo in Cameroon, (ii) the Ewondo and Xhosa tribes also share similar cultural and rite of passage practices such as the ritual of male circumcision and the burial of the umbilical cord or placenta of new-born as part of welcoming the new-born and introduction to the ancestors; (iii) and religious beliefs such as the ‘cult of ancestry’ and reincarnation are common amongst Bantu-speakers. Despite these and many other shared cultural, linguistic and anthropological attributes, the present data further support the notion that Bantu-speakers from Central and West Africa are no more genetically similar to those in Southern Africa, as previously illustrated with differential prevalence of HIV resistant genes amongst SSA populations [39, 40] and in this paper with malaria-associated variants. Given the vast genetic diversity within the continent and amongst any two SSA populations, the present research further emphasises the need to redefine the classifications of various groups in Africa by region-defined genomic attributes, as this approach could better serve Genomic medicine practice, as opposed to the classical ethno-linguistic population classification approach; the modest data presented here illustrate that at the genetic levels, Bantu is not equal to Bantu.

### SCD *β*-globin haplotype: insights into the migration of Southern African blacks

The third question of this study was to investigate the degree of conservation of the five SCD haplotype-conferring loci in populations both largely unaffected by the disease and void of the environmental pressure of malaria. The most apparent, although not surprising result, was the high frequency of the atypical haplotypes in all the study cohorts leading to the hypothesis that in such populations, the five loci of the *β*-globin gene cluster may be under less evolutionary pressure to remain conserved. This could be due to several reasons; there is no apparent clinical benefit to retaining an otherwise unfavorable haplotype in the absence of malaria and potentially its strongest environmental positive selector, malaria. Furthermore, this could be as a result of genetic drift and recombination at the *β*-globin gene cluster. The next frequent haplotype in all study cohorts was the Benin form, suggesting that the Southern African Bantu-speakers migrated southwards, post-occurrence of the *HbS* mutation and is consistent with their West African origins [16]. The data showing the classification of the *HbS* haplotypes in these Southern African populations and the degree of similarity among the haplotype distributions in South Africa, Zimbabwe and Malawi is novel. The data suggest some insight into the evolutionary dynamics at the *β*-globin loci with regard to recombination of the classical *HbS* haplotypes and expansion of the atypical form in malaria-devoid regions in Africa.

Indeed, the result confirms to anthropological data detailing the most significant events of the geographic expansion of the Bantu Niger-Kordofanian-speakers out of Cameroon and Nigeria [17, 18]. It was previously hypothesised that the migration path was first through rainforest equatorial Africa and later into Eastern and Southern Africa. This is supported by the widespread distribution of Bantu-related linguistic groups and the presence of Niger-Kordofanian genetic ancestry in many African populations. However, the present result with a prevalent Benin haplotype and very low Bantu haplotype that is characteristic of SCD patients from Central and West Africa [16] could be due to a myriads of possible reasons that remain to be investigated: (i) the migration through East Africa of modern Southern African Bantu-speaking populations was transient with limited admixture with populations found locally in East Africa; (ii) some Bantu haplotypes may have been lost during recombination events at the *β*-globin gene locus potentially leading to the expansion of the highly prevalent atypical form; (iii) during the early migration events through the equatorial rainforests, the migrating populations from Central, East and West Africa encountered largely unoccupied regions, therefore expanding the Benin, Cameroon and Senegal haplotypes; (iv) the Bantu haplotype could be a recent haplotype of SCD, only recently expanding in Central Africa and subsequently in some parts of North and South America through slave trade; and lastly (v) the continuous socio-economically motivated migration from Central, East and West Africa into Southern African countries could have led to the relatively higher frequencies of the Benin, Cameroon and Senegal haplotypes although unlikely as this has become a significant migration phenomenon only in the past two to three decades. Beyond the concept of the dissociation between genetic background and ethno-linguistic attributes and classifications, the present data also complements previous studies on migrations of Southern African populations from West and/or Central Africa [38].

A limitation of the present study includes the number and the selection of malaria-associated variants selected. Given that the two SNPs in *HBB* and ABO are only weakly linked to the causative SNPs in the Ghana [21], it is likely that they are poor tags for the known causative SNP in the South African population. In addition, future studies should investigate the full distribution of ‘atypical’ haplotypes among *HbAA* and *HbAS* individuals from both malaria endemic and non-endemic areas, to have a full profile of *HBB* haplotypes in Africa.

## Conclusion

These selected malaria-associated variants in SSA suggest differences among ‘Bantus’ from various part of Africa, and emphasise the evidence of the dissociation between genetics, anthropology and culture. The present study also showed a relatively prevalent Benin haplotype, which is mostly found in West Africa, among Southern African Blacks and very low Bantu haplotype, which could suggest a major migration route, of Southern Africa Bantu, along the African west coast, post-occurrence of the sickle cell mutation. The data are indicative of the importance of the inclusion of Southern African populations when studying the age and origin of the HbS mutation, that remains to be fully elucidated [16]; Future studies should include Khoi and San populations, some of which may not have been exposed to malaria, and sequence around regions where our present results indicate LD with functional mutation to unravel novel candidates. Furthermore, these data also provide additional genetic evidence indicating the independent and continuous waves of migration of West and East African Bantu-speaking groups into Southern Africa. Beyond the data presented here, the high proportion of atypical haplotypes in Southern African populations, together with the data from diverse populations on the African continents could suggest various level of genetic diversification of African populations, whether attributable to recent and/or more ancient admixture, that did not probably result from a single North to South migration path nor a specific era, but rather through several independent and associated, multi-directional migration events [41–43]. It can be anticipated that modern-day continuous immigration, will further reinforce the African genomic diversity, by allowing the redistribution of gene pools previously restricted to specific geographical location, such as malaria-related mutations, across the continent.

## Supplementary material

For supplementary material accompanying this paper visit http://dx.doi.org/10.1017/gheg.2017.14. click here to view supplementary material
