# Supplementary material for: Beta-globin gene haplotypes and selected Malaria-associated variants among black Southern African populations
Source: Glob Health Epidemiol Genom. 2017 Nov 27;2:e17. doi: 10.1017/gheg.2017.14 (PMC5870409; doi:10.1017/gheg.2017.14)
Supplement: Supplementary file 1 [file S2054420017000148sup001.zip › Supplementary Tables and Figures Captions.docx]

# **List of Supplementary Tables and Figures**

**Table S1.** Restriction endonuclease cutting patterns that represent each of the five β-globin gene haplotypes.

**Table S2.** Restriction endonuclease cutting patterns that represent each of the five most common Atypical β-globin gene haplotypes.

**Table S3.** Frequency of various forms of Atypical β-globin haplotypes in Southern African populations.

**Table S4.** Haplotypes frequencies at *rs334* with known alleles A/T, encoding the Hb A form of (adult) hemoglobin and the sickling form of hemoglobin, Hb S.

**Figure S1.** Proxy Linkage Disequilibrium (LD) and functional variants for malaria associate SNP, *rs372091*: Plot displays variants that are in LD to *rs372091*. Proxy LD obtained using (A) YRI (as proxy population for West Africa) and (B) LWK (as proxy population for East Africa) from 1000 Genomes data. Proxy LD was computed using Ldlink [46].

**Figure S2.** Proxy Linkage Disequilibrium (LD) and functional variants for malaria associate SNP, *rs8176703*: Plot displays variants that are in LD to *rs8176703*. Proxy LD obtained using (A) YRI (as proxy population for West Africa) and (B) LWK (as proxy population for East Africa) from 1000 Genomes data. Proxy LD was computed using Ldlink [46].

**Figure S3.**  Proxy Linkage Disequilibrium (LD) and functional variants for malaria associate SNP, *rs2334880*: Plot displays variants that are in LD to *rs2334880*. Proxy LD obtained using (A) YRI (as proxy population for West Africa) and (B) LWK (as proxy population for East Africa) from 1000 Genomes data. Proxy LD was computed using Ldlink [46].

**Figure S4.** HbS allele frequency in Africa (adapted from [14])

# Reference

46. **Machiela MJ , Chanock SJ.** LDlink a web-based application for exploring population-specific haplotype structure and linking correlated alleles of possible functional variants. *Bioinformatics*. 2015 Jul 2. PMID:26139635.
