# Supplementary material for: Beta-globin gene haplotypes and selected Malaria-associated variants among black Southern African populations
Source: Glob Health Epidemiol Genom. 2017 Nov 27;2:e17. doi: 10.1017/gheg.2017.14 (PMC5870409; doi:10.1017/gheg.2017.14)
Supplement: Supplementary file 1 [file S2054420017000148sup001.zip › Supplementary Table S3.docx]

**Supplementary Table S3.** Frequency of various forms of Atypical β-globin haplotypes in Southern African populations

|  | *Haplotype frequency by population n (%)* | | | |
| --- | --- | --- | --- | --- |
| Atypical types | ***South Africa*** | | ***Zimbabwe*** | ***Malawi*** |
| I | 17 (32.1) | 16 (38.1) | | 14 (38.9) |
| II | 13 (24.5) | 10 (23.8) | | 9 (25.0) |
| IIi | 4 (7.5) | 5 (11.9) | | 6 (16.7) |
| iv | 4 (7.5) | 2 (4.8) | | 3 (8.3) |
| v | 4 (7.5) | 3 (7.1) | | 2 (5.6) |
