# Supplementary material for: Beta-globin gene haplotypes and selected Malaria-associated variants among black Southern African populations
Source: Glob Health Epidemiol Genom. 2017 Nov 27;2:e17. doi: 10.1017/gheg.2017.14 (PMC5870409; doi:10.1017/gheg.2017.14)
Supplement: Supplementary file 1 [file S2054420017000148sup001.zip › Supplementary Table S2.docx]

**Supplementary Table S2.** Restriction endonuclease cutting patterns that represent each of the five most common Atypical β-globin gene haplotypes

|  |  |  | *Enzymes* |  |  |
| --- | --- | --- | --- | --- | --- |
| Atypical TYPES | ***XmnI (5′Gϒ)*** | ***HindIII (Gϒ)*** | ***HindII (Aϒ)*** | ***HincII (3′ᵠβ)*** | ***HinfI (5′β)*** |
| I | - | - | - | + | + |
| II | - | + | - | - | + |
| IIi | - | + | - | + | + |
| iv | - | - | - | - | + |
| v | - | + | - | + | - |
