# Supplementary material for: Beta-globin gene haplotypes and selected Malaria-associated variants among black Southern African populations
Source: Glob Health Epidemiol Genom. 2017 Nov 27;2:e17. doi: 10.1017/gheg.2017.14 (PMC5870409; doi:10.1017/gheg.2017.14)
Supplement: Supplementary file 1 [file S2054420017000148sup001.zip › Supplementary Table S1.docx]

**Supplementary Table S1.** Restriction endonuclease cutting patterns that represent each of the five β-globin gene haplotypes

|  | *enzymes* | | | | |
| --- | --- | --- | --- | --- | --- |
| haplotypes | ***XmnI (5′Gϒ)*** | ***HindIII (Gϒ)*** | ***HindII (Aϒ)*** | ***HincII (3′ᵠβ)*** | ***HinfI (5′β)*** |
| Arab-Indian | + | + | - | + | - |
| Bantu/CAR | - | + | - | - | - |
| Benin | - | - | - | + | - |
| Cameroon | - | + | + | + | + |
| Senegal | + | + | - | + |  |
