# Supplementary figures and images for: Beta-globin gene haplotypes and selected Malaria-associated variants among black Southern African populations
Source: Glob Health Epidemiol Genom. 2017 Nov 27;2:e17. doi: 10.1017/gheg.2017.14 (PMC5870409; doi:10.1017/gheg.2017.14)

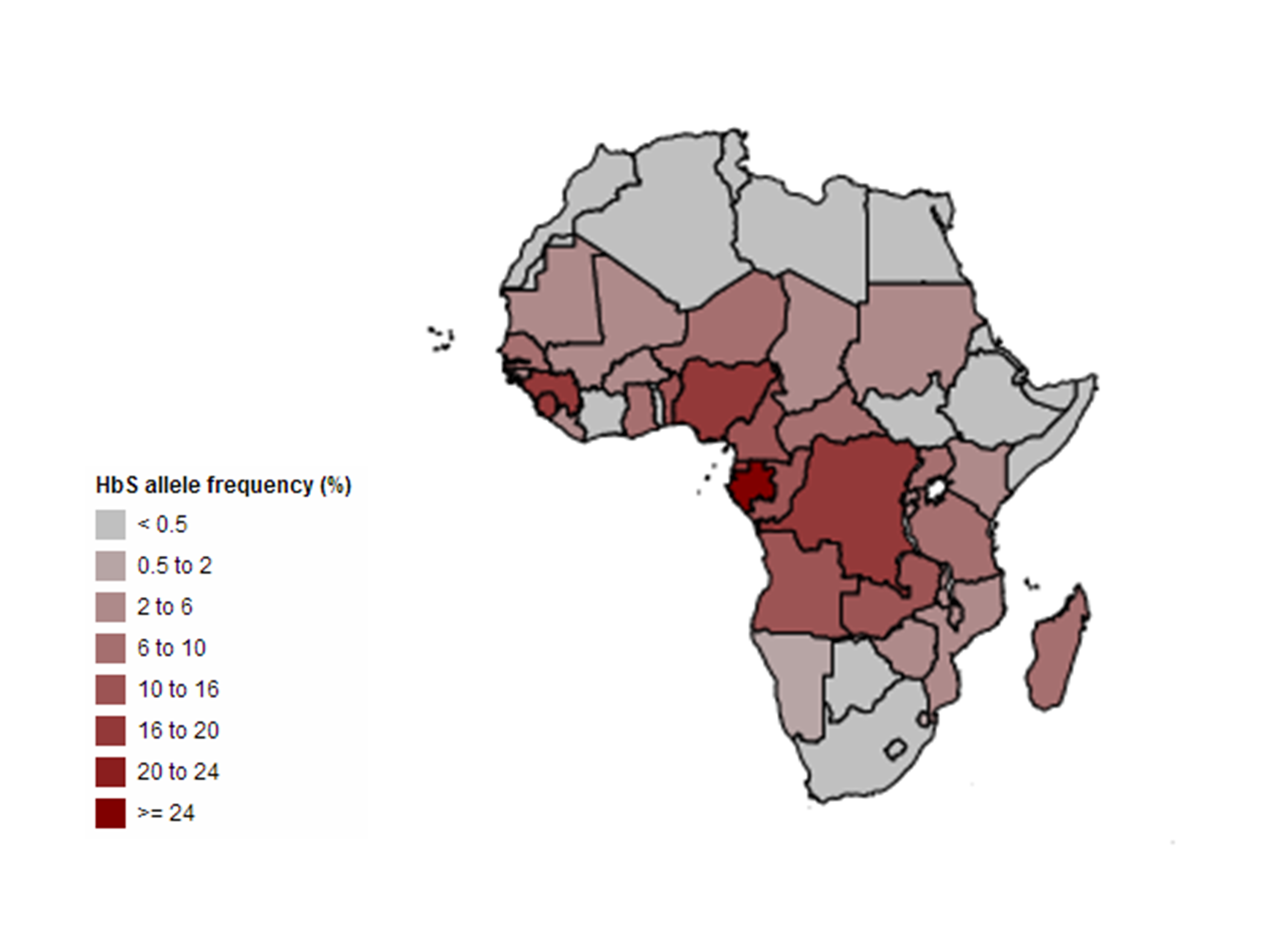

Supplement: Supplementary file 1 [file S2054420017000148sup001.zip › Figure S4.tif]

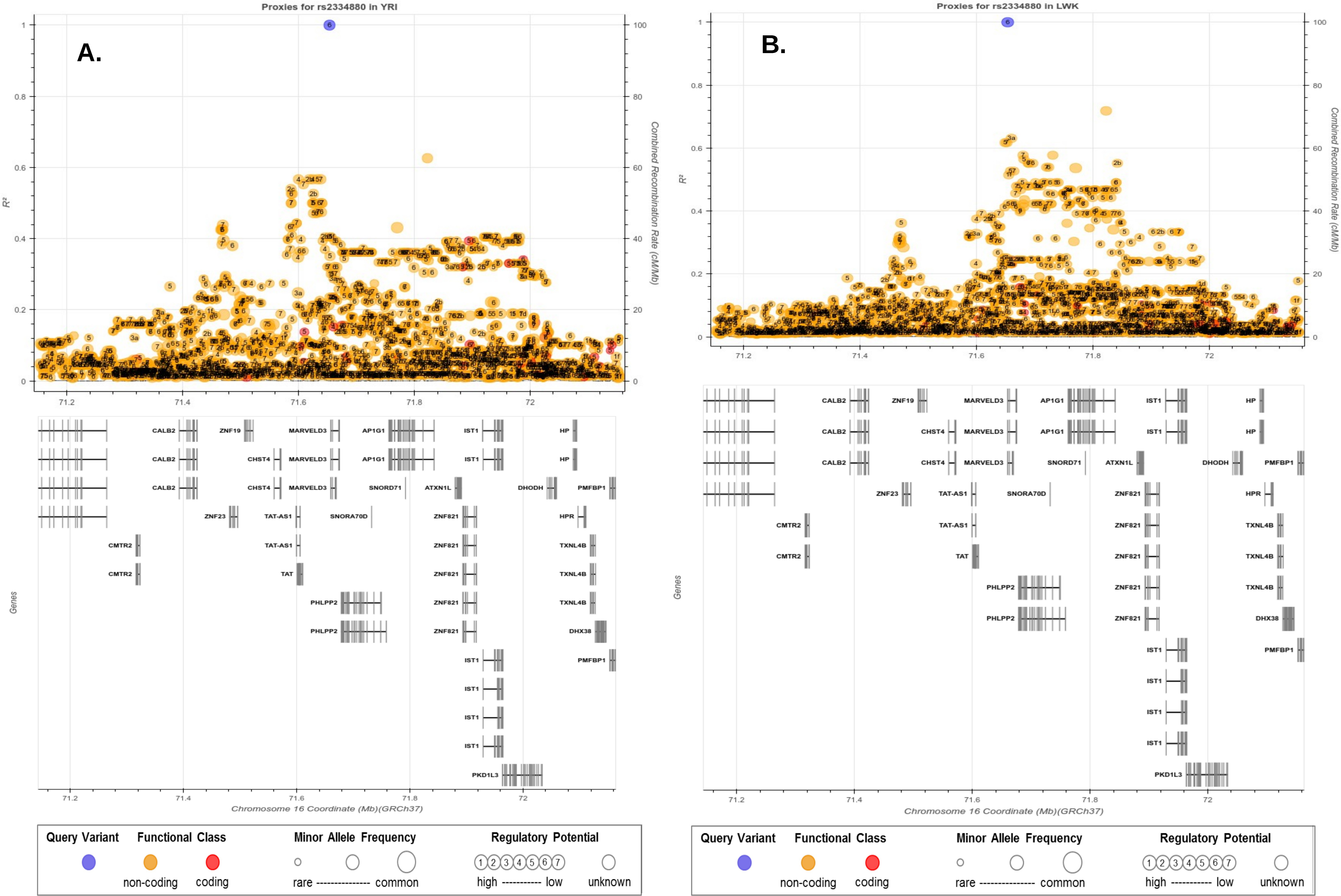

Supplement: Supplementary file 1 [file S2054420017000148sup001.zip › Figure S3.tiff]

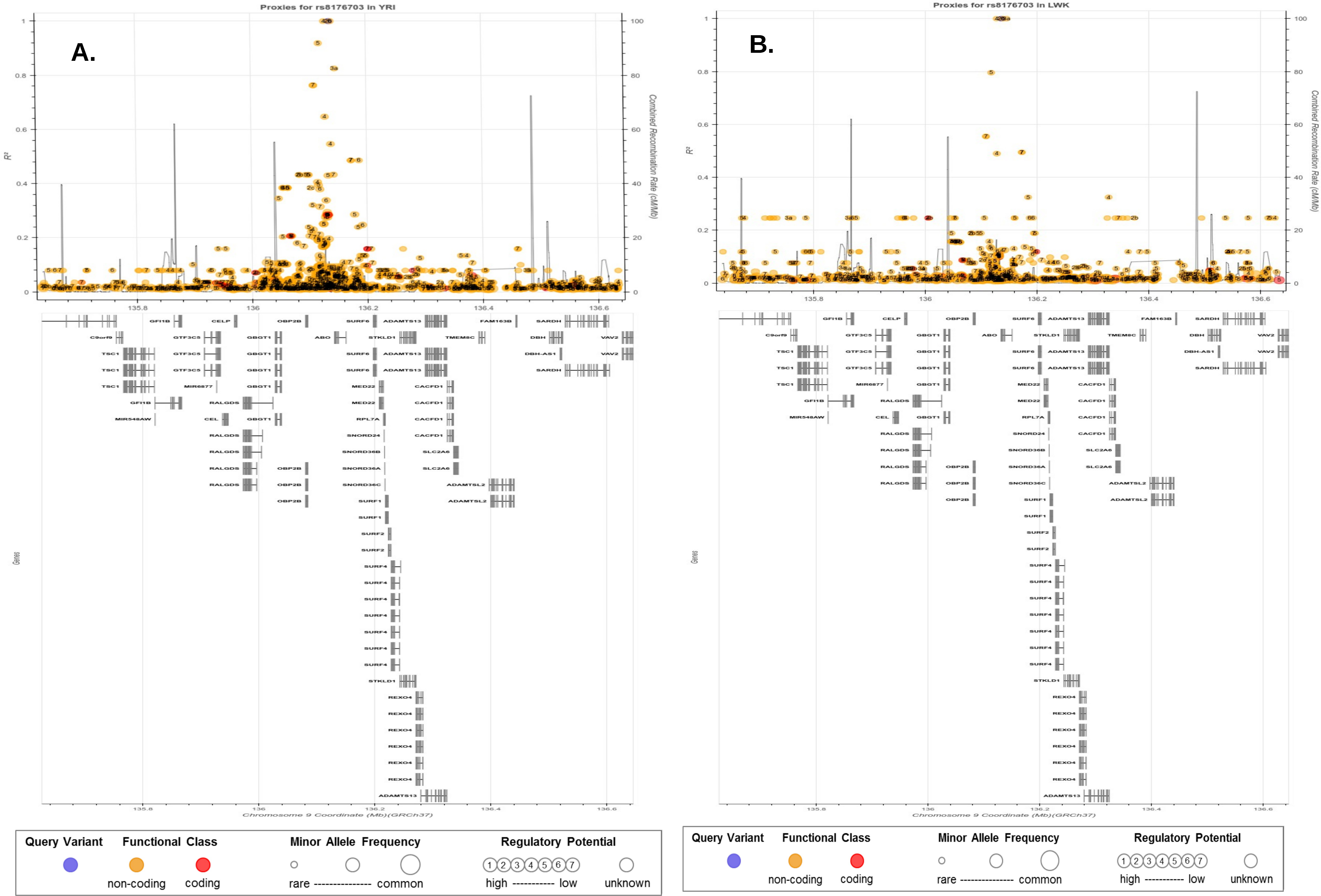

Supplement: Supplementary file 1 [file S2054420017000148sup001.zip › Figure S2.tiff]

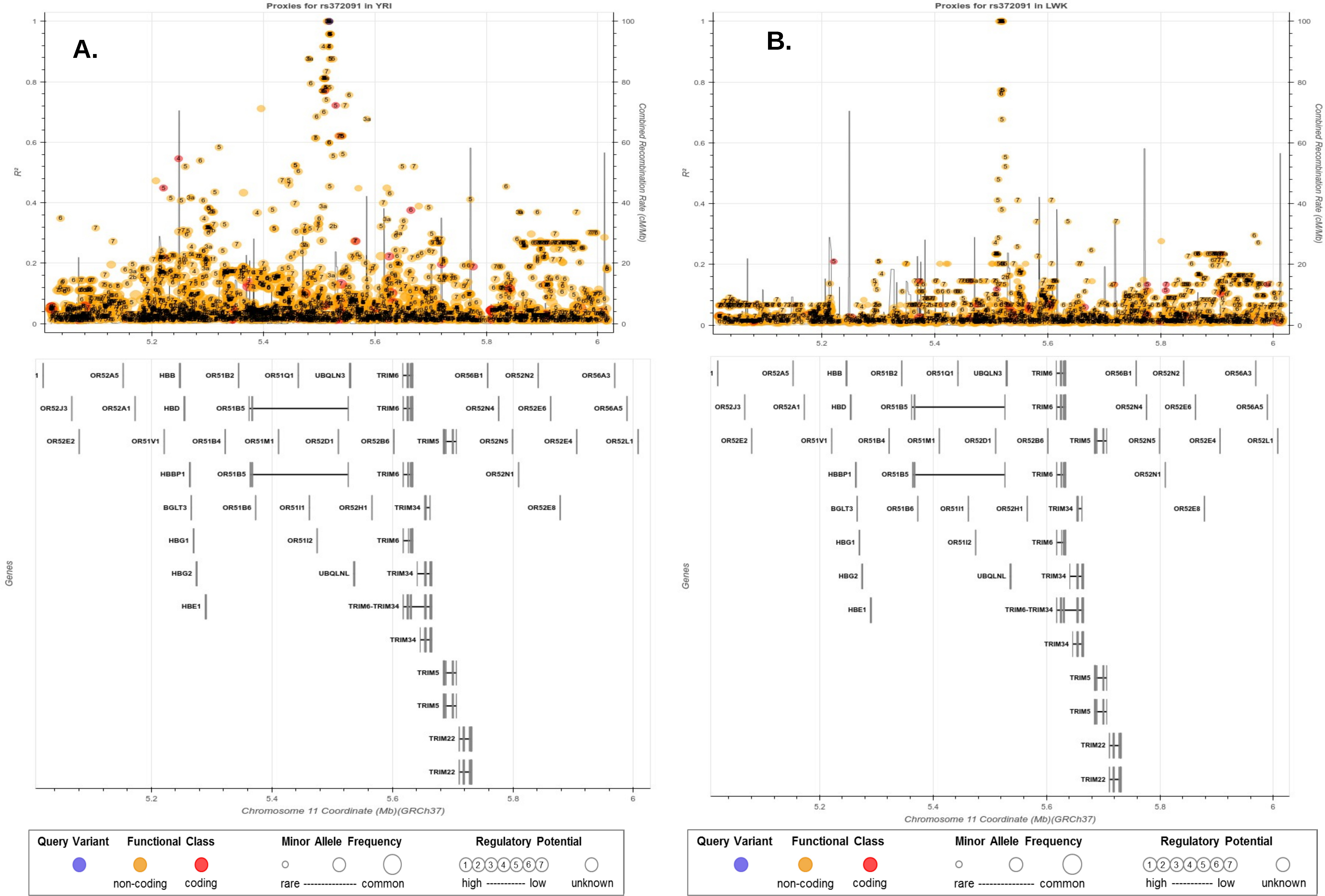

Supplement: Supplementary file 1 [file S2054420017000148sup001.zip › Figure S1.tiff]
